# Rheological and structural properties of enzyme‐induced gelation of milk proteins by ficin and *Polyporus badius*

**DOI:** 10.1002/fsn3.553

**Published:** 2017-12-15

**Authors:** Reihaneh Shabani, Seyed‐Ahmad Shahidi, Ali Rafe

**Affiliations:** ^1^ Department of Food Science and Technology Collage of Agriculture and Food Science Ayatollah Amoli Branch Islamic Azad University Amol Iran; ^2^ Department of Food Processing Research Institute of Food Science and Technology (RIFST) Mashhad Iran

**Keywords:** Ficin extract, induced gelation, microstructure, *Polyporus badius*, rheology

## Abstract

The rheological and microstructural characteristics of ewes’ milk curd obtained by coagulating with milk‐clotting enzymes, including ficin extract and *Polyporus badius* were evaluated. The gelation of milk was examined by small amplitude oscillatory shear measurements (SAOS). Different concentrations of ficin and *P. badius* extracts (1, 3, and 5%) were utilized to coagulate milk proteins. The ewes’ samples containing ficin and mushroom enzymes were heated from 25 to 45°C at a heating rate 1°C/min and kept for 30 min. Then, the curds were cooled down to 25°C with the same heating rate. The ficin extract could induce stronger gels at 45°C and 5% ficin. Similar results were also found for 5% *P. badius* extract and incubation at 45°C. However, *P.badius* gels achieved a network with more viscous characteristics and had a softer texture than ficin gels. Therefore, it may be concluded the induced gels with mushroom had higher moisture and lower protein contents, which related to the high proteolytic activity of *P. badius*. The microstructure survey showed that the mushroom‐induced gel had a more compact structure. By increasing enzyme concentration, both gels showed a coarser and more compact protein network. Whereas, the *P. badius* gels had more fusions and folds which indicate the greater proteolysis occurred during gelation and there was greater breakdown of protein. Our findings suggest the application of ficin and *P. badius* enzymes to develop a novel procedure to coagulate milk proteins and providing new structures in food systems.

## 1 INTRODUCTION

One of the most complex and important biochemical events in milk gelation is proteolysis. In most cases, caseins are hydrolyzed by enzymes of coagulant and to some extent by milk enzymes. In cheese making, the rennet was chiefly used to coagulate milk proteins. The quality of rennet enzymes has a great effect on the process of the curd formation as well as the organoleptic characteristics of the dairy product. The chymosin plays a vital role in the process and it is liable to hydrolysis the Phe105‐Met106 bond in κ‐casein into para‐κ‐casein and glycolmacropeptide. Due to the reduced supply and expensiveness of rennet, ethical factors, diet or ban on recombinant calf rennet, the demand for finding alternative sources of rennet is growing (Cavalcanti, Teixeira, Lima Filho, & Porto, 2004). In contrary, microbial enzymes have been used widely in cheese making in recent decades (Bukhalo, 1988; Gudkov, 2004), but the unbalanced proteolytic activity of the enzymes disintegrates proteins into bitter peptides and accumulate high amount of hydrophobic peptides with intermediate size (Habibi Najafi & Lee, 1996; He et al., 2011; Raposo & Domingos, 2008).

The ease of accessibility, purification, inexpensiveness, and satisfactoriness of plant proteases for vegetarian people as well as their similarity to chymosin as they are aspartic proteinases, made them more preferable than other natural coagulants (Tavaria, Sousa, & Malcata, 2001). Although, the plant coagulants due to low yield, off‐flavor, and soft texture are commonly inappropriate in cheese making which limit their application in the dairy industry, but they have some advantages over other coagulants such as activity in a wide range of pH, temperature, and presence of organic compounds (Sharma, Kumari, & Jagannadham, 2009). In recent decades, there is unremitting challenges to find new plant proteases and know their milk gelling activities. The literature survey showed that various plant proteases such as latex of fig in Teleme (Mallatou, Pappa, & Boumba, 2004; Pappa, Kandarakis, & Mallatou, 2007), flowers of cardoon in Serra da Estrada and Serpa, two popular Portuguese cheeses (Chazarra, Sidrach, Lopez‐Molina, & Rodrıguez‐Lopez, 2007; Chen, Agboola, & Zhao, 2003; Roseiro, Barbosa, Ames, & Wilbey, 2003; Roseiro et al., 2003; Silva, Barros, & Malcata, 2002), paw paw (Bornaz et al., 2010), pineapple (Cattaneo, Nigro, Messina, & Giangiacomo, 1994) and castor oil seeds have been applied in milk coagulation. The studies showed that some mushrooms have the ability to use in milk gelation and it was found that *basidiomycetes* have high enzyme activity like as rennet and some of them have applied in practical applications (Shamtsyan, Dmitriyeva, Kolesnikov, & Denisova, 2014).

*Polyporus badius* is a member of *Polyporaceae* family belonging to the *Basidiomycota* (Borhani, Ali‐Mosazadeh, & Badalyan, 2014; De, 1997; Krüger, Petersen, & Hughes, 2006). Gelation of ewe's milk in the presence of *P. badius* is a process utilized for many years in northern of Iran. The curd is traditionally made using raw ewe milk, where no starter is added. The autochthonous product has been mainly consumed with cooked rice and it has called as “*Halvi”* or “*Halbi”*. Halvi is soft and spongy curd containing high level of fat without any supplementary process such as ripening. It is believed to have high nutritional value and performance characteristics. Since, wild *P. badius* is scare; fig latex is also used to produce Halvi.

The objective of the current work was to evaluate the rheological and structural properties of the gelation of ewe's milk using ficin and *P. badius* enzymes as novel proteases. Indeed, scientific information on the rheological behavior of milk gel can be utilized by industrial managers in deciding the proper way to have a different texture in the dairy industry.

## 2 MATERIALS AND METHODS

### 2.1 Materials

Dried figs were purchased from local markets of Sari (Mazandaran province, Iran). The edible wild mushroom, that is, *P. badius* was obtained from Mazandaran forests. It was authenticated by Dr. Riahi (Shahid Beheshti University, Tehran, Iran) as *P. badius* using relevant text books and literature published on mushroom species growing in Iran as well as morphological and anatomical comparisons. The mushrooms were dried at ambient temperature and kept in the refrigerator (4°C) till the experiments. All the ingredients were of analytical grade and purchased from Sigma Aldrich (St. Louis, MO).

### 2.2 Curd preparation

**Figure 1:**
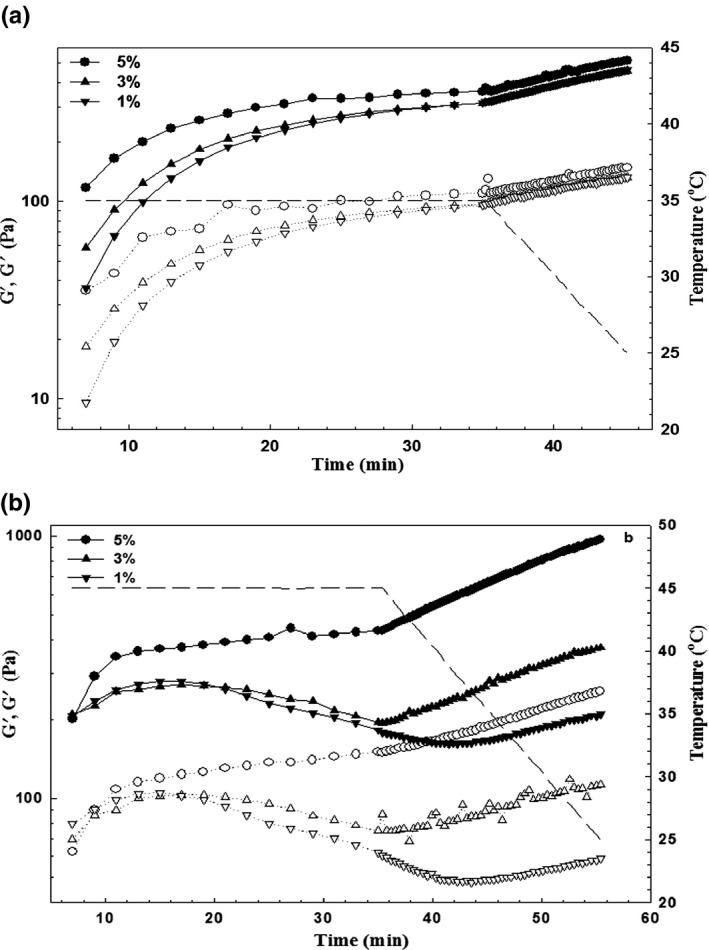
Effect of different concentrations of ficin extract on the gelation of ewe's milk. 35°C (a) and 45°C (b). G′ and G′′ are presented with filled and open symbols, respectively

Raw ewes’ milk was supplied from farms of Amol (Mazandaran, Iran) and it was transferred to laboratory using cold box system. The quality characteristics of ewes’ milk, including chemical composition were determined using Milkoscan (model 134 A/B N, Foss Electric, Denmark). The pH was determined using a pH‐meter (model 632 Metrohm, Switzerland) and the ash content was measured by standard analytical methods (AOAC, 2005). The ewes’ milk was pasteurized by a serrated plate heat exchanger at the 78°C for 1 min and then cooled and divided into two groups 35 and 45°C. The dried mushrooms were cut into 1–2 mm specimen and 10 g of them poured into distilled water (60 ml) and maintained overnight at 4°C to complete the hydration. Prior to inoculation, the mushroom extract was released by pressure. Then, the mushroom extract at different concentrations 1, 3, and 5% were inoculated to the milk groups (35 and 45°C). For the figs, appropriate amount of fig (1, 3, and 5%) was located in the filter cloth and transferred into the ewes’ milk with temperatures 35 and 45°C. In that case, they were massaged in milk for 2 min. Then, the samples of inoculated with mushroom/ficin extract were transferred into the incubator with the same temperature for 40 min. After the coagulation, the samples were cooled down to the room temperature and preserved in the refrigerator (4°C) till further experiments.

### 2.3 Chemical composition

Coagulated samples of ewes’ milk were analyzed in triplicate for moisture, fat, pH, titratable acidity (as percentage of lactic acid), and total nitrogen by the methods described by Ardö and Polychroniadou (1999). The moisture and fat content of curds were determined using a moisture analyzer (Sartorius Ltd., Epsom, UK) and Gerber method (AOAC, 2005), respectively. For pH measurement, the grated cheese samples (10 g) mixed with equal quantities of distilled water (10 ml) and the pH of dispersion was measured using pH‐meter (Ardö & Polychroniadou, 1999). Titratable acidity of curd samples was determined by Dornic method (Robinson & Wilbey, 1998). Cheese samples were analyzed for ash content by the dry ash method at 550°C (AOAC, 2005). The nitrogen content, sugar, and inverted sugar were also determined using AOAC methods.

### 2.4 Rheological assays

A controlled stress/strain rheometer (Paar Physica rheometer, MCR 301, Anton Paar GmbH, Germany), which was fitted to bob and cup geometry with medium sensitivity, was applied to perform small‐deformation oscillatory measurements. Temperature was controlled by a Peltier system (Viscotherm VT2, Phar Physica) to control temperature fast and precisely. According to the similar works on the rheology of cheese, the weight sample has the great effect on the results, and therefore the samples were matched on a weight basis (Wium and Qvist, 1997, 1998; Tunick et al., 1990). In order to determine the gelling conditions, the ewes’ milk samples were poured and inoculated with *P. badius*/ficin extracts in situ in the cup of rheometer at 25°C and equilibrated at least 5 min prior to the experiment. Following inoculation, the samples were kept at 35 and 45°C for 30 min and then, they were cooled down to 25°C at the rate of 1°C/min. The temperature sweep was performed at a frequency of 1 Hz and 0.5% strain. Immediately, the frequency sweep (10^‐2^–10^2^ Hz) was applied to exhibit the type of gelation and gelling characteristics. All the rheological experiments were performed in duplicate and in the linear viscoelastic region.

### 2.5 Microstructure

The microstructure of gels induced by enzymes of ficin and *P.badius* was measured by scanning electron microscopy (SEM) based on our previous works (Rafe, Razavi, & Farhoosh, 2013; Rafe, Vahedi, & Ghorbani Hasan‐Sarei, 2016). In brief, the curd samples were cut into small pieces with a sharp razor and fixed in 2.5% glutaraldehyde for at least 1 hr. Then, they were rinsed three times in cacodylate buffer, and postfixed in a 1% buffered OsO_4_ for 1 hr. The dehydration of fixed sample was accomplished in a graded series of ethanol. Then, the samples were freeze dried and at least three dried samples of each gel were fractured mounted on aluminum stubs, and coated with gold in a sputter coater (Balzers, type SCD 005; Baltec Inc., Switzerland). After evaluation 10–15 regions of each sample, the images of typical structures at 1,000, 5,000, and 10,000 magnifications were obtained. The morphology of the gels was observed under a scanning electron microscope (LeQ1450VP, Vereinigte Papierwarenfabriken GmbH, Feuchtwangen, Germany) with an accelerating voltage of 26 kV.

### 2.6 Statistical analysis

Rheological data and graphs were analyzed by Rheoplus software (version 3.40 Anton Paar GmbH, Germany) and Sigmaplot (version 8.0; Jandel Scientific, Corte Madera, CA, USA), respectively. The Tukey multiple‐comparison test was used as a guide for pair comparisons of treatment means. The level of significance of differences between treatments was considered at *p* < 0.05.

## 3 RESULTS AND DISCUSSION

### 3.1 Chemical composition

The chemical composition of dairy gels in the presence of ficin and *P. badius* is presented in Table 1. The pH, fat and moisture content of both gels were similar, but the proteins, solid nonfat (SNF) were significantly different. Indeed, the presence of enzymes of *P. badius* and ficin were significantly influenced the protein contents of the gel, as shown in Table 1 (*p* < 0.05). The pH of the curd at the end of the incubation was also affected by the type of enzymes and the procedure. pH at the end of incubation was 5.2 which are in agreement with the previous works (Dubrova Mateva, Naletoski, & Palasevski, 2008).

**Table 1:** Chemical composition of dairy gels in the presence of ficin extract and *P. badius* a

Type of coagulant	pH	Moisture	Fat	Protein	Total carbohydrate	Succors	Ash
Ficin extract	5.4 ± 0.1	81.55 ± 0.10	4.3 ± 0.15	6.4 ± 0.20	56.3 ± 1.4	53.4 ± 1.6	0.90 ± 0.05
*P. badius*	5.3 ± 0.1	83.95 ± 0.20	4.4 ± 0.14	5.3 ± 0.10	40.6 ± 1.8	39.5 ± 1.3	0.80 ± 0.04

a The mean values from two batches of gel with three replicates per analysis. The measurement was presented in w/w%.

### 3.2 Induced gelation of ewes’ milk in the presence of ficin

Since, the curd has a soft behavior and showed the viscoelastic properties; the small‐strain rheological test can be applied to discover its rheological behavior. The small‐strain test should be conducted in the linear viscoelastic region, defined as where stress is linearly proportional to strain at a given strain rate (Barnes, Hutton, & Walters, 1989; Steffe, 1996).

The effect of ficin extract at different concentrations (1, 3, and 5%) on the storage (G′) and loss modulus (G′′) during incubation at 35 and 45°C for 30 minutes and cooling down to 25°C is presented in Figure 1. It can be seen that G′ of ewes’ milk containing ficin extract was increased sharply initially at an incubation period at 35°C and becomes monotonous at the end of incubation. Although, increase in G′ at 45°C was not found for 1 and 3% ficin extract, which may be attributed to the lower amount of ficin and its degradation. On the other word, ficin enzymes were significantly affected by heating at 45°C and may be denatured more at this temperature. Whereas, the gelation of milk proteins at 35°C have occurred in the presence of ficin extract. Ficin has the proteolytic activity in which cysteine protease acts on bonds involving uncharged and/or aromatic amino acids. According to Akar and Fadiloglu (1999) ficin (EC 3.4.22.3) contains two groups of proteolytic enzymes, including a high milk clotting enzyme, low proteolytic activity, and higher a specific proteolytic effect. The elasticity of gels at the end of incubation at 35°C were approached to 316 ± 2.2, 319 ± 3.4, and 375 ± 3.1 Pa, which were more than G′ values for incubation at 45 °C except 5% ficin extract (435 Pa). In addition, G′ value was higher than that of G′′ at the outset and throughout the experiment, revealed the association of proteins. The elasticity of gels was improved by cooling at all ficin concentrations and temperatures. It was not observed any statistical significant difference between different concentrations of ficin extract at 35°C (*p* < 0.05). A comparison between different incubation temperatures showed that the storage modulus of sample containing 5% ficin was improved to the more value by incubation at 45°C (~970 Pa). Therefore, it can be concluded that incubation at 45°C with more ficin enzyme can develop stronger gels than all the other ficin concentrations.

### 3.3 Induced gelation of ewes’ milk in the presence of mushroom enzymes

**Figure 2:**
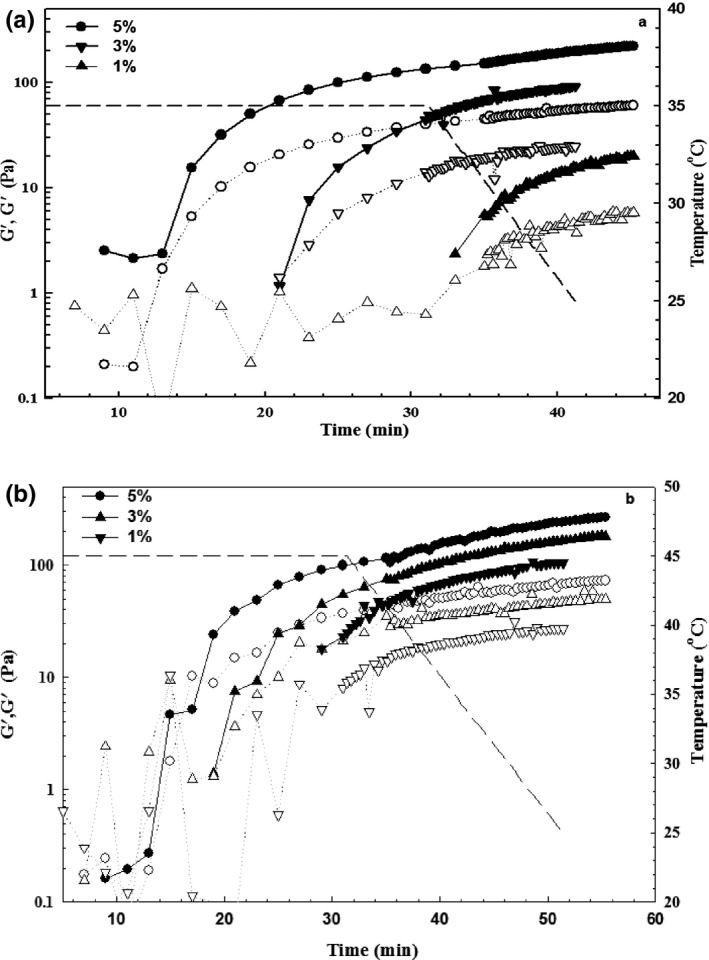
Effect of different concentrations of mushroom enzymes on the gelation of ewe's milk. 35°C (a) and 45°C (b). G′ and G′′ are presented with filled and open symbols, respectively

The effect of mushroom enzymes at different concentrations (1, 3, and 5%) on the G′ and G′′ during incubation at 35 and 45°C for 30 min and cooling down to 20°C is presented in Figure 2. The G′ value of ewe's milk was increased sharply at both incubation temperatures and enhanced more by cooling. Since, the gelation of milk protein was occurred more rapidly at higher mushroom enzyme concentrations at both incubation period; it can be easily understood that the concentration of enzymes was profoundly affected the gelation of milk protein. On the other hand, the milk proteins need more time to aggregate at lower concentrations of mushroom enzymes.

The gelling point (T_gel_) is determined by extrapolating the rapidly rising G′ values back to the temperature axis, which can be determined as the temperature when an infinite network occurs in the sample and it is time at which tan δ is independent of frequency (Rafe et al., 2012, Tunick, 2011; Hsieh, Regenstein, & Rao, 1993). Surprisingly, the gelling point of specimen at the similar concentration of mushroom enzyme was not affected by the incubation temperatures (Figure 2). The G′ value at the end of incubation at 35°C were approached to 5.2 ± 0.5, 48.4 ± 1.3, and 150 ± 2.4 Pa (1, 3, and 5%), which more than the incubation at 45°C at all concentrations. Thus, it can be distinguished that mushroom enzymes were more disintegrated at 45°C. The cooling period had a significant effect on the elasticity of gels and G′ value was improved at all concentrations and temperatures. A comparison between samples at different incubation temperatures showed that G′ value of the sample containing 5% enzyme was improved to more value by incubation at 45°C (~226 Pa). Therefore, it can be concluded that incubation at 45°C with high amount of *P. badius* enzyme can develop stronger gels than the others. Since, *P. badius* gels obtained a network with more viscous property and had a softer texture than that of ficin gels, it may be concluded that induced gels with mushroom have a higher moisture and lower protein contents, related to the high proteolytic activity of *P.badius* as well as the lubricant effect of whey protein among the casein micelles (Fox et al., 2000).

### 3.4 Mechanical assay of ficin and mushroom enzymes on gelation

**Figure 3:**
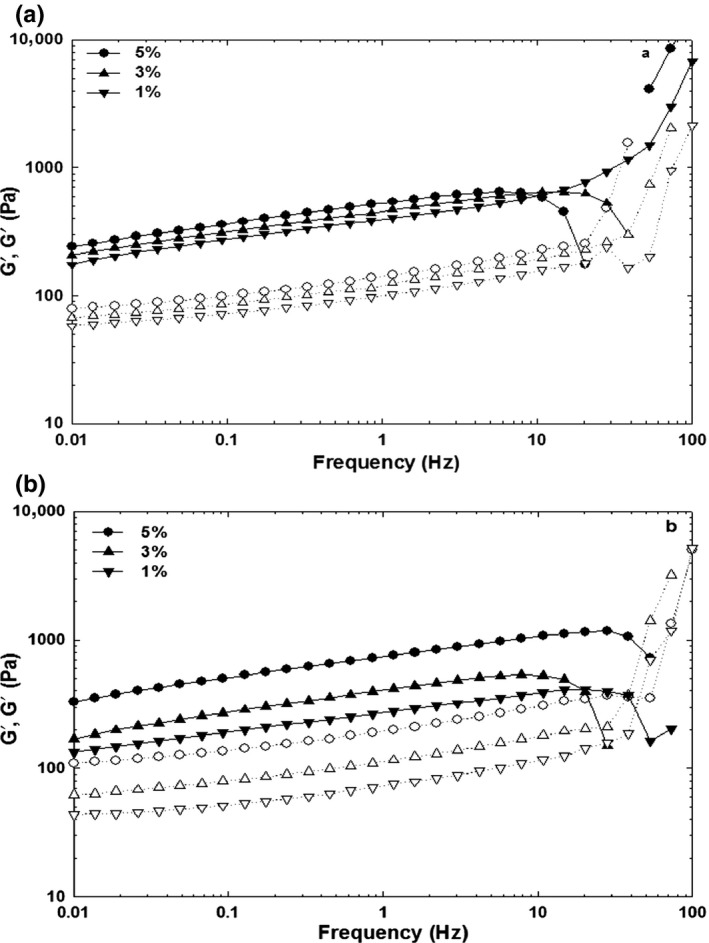
Typical mechanical spectra for ficin‐induced gelation of ewe's milk at different incubation temperature 35°C (a) and 45°C (b). G′ and G′′ are presented with filled and open symbols, respectively

**Figure 4:**
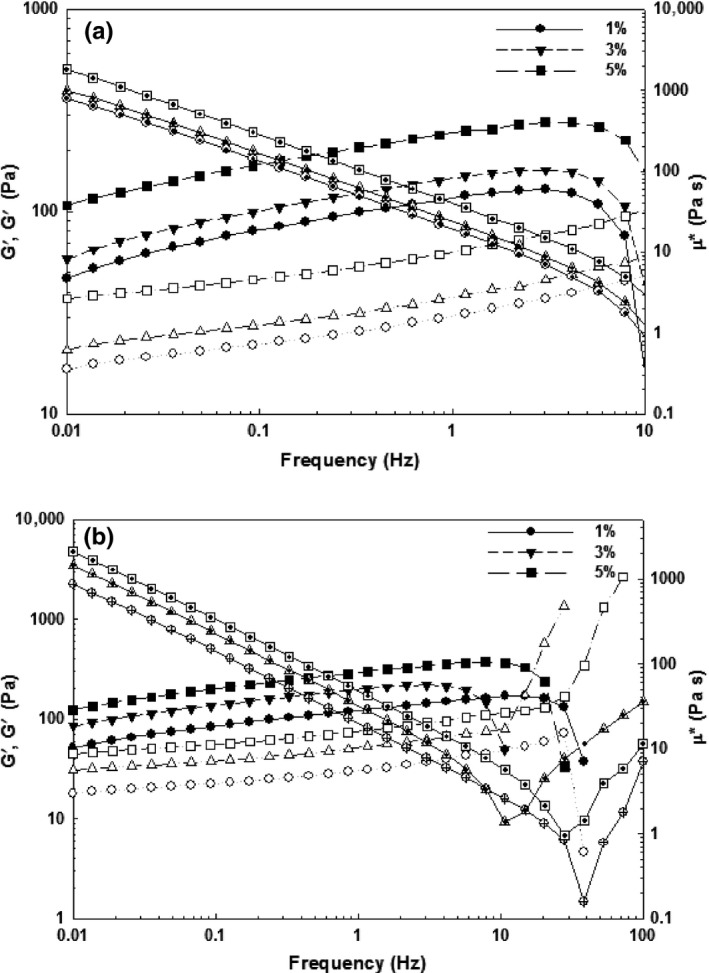
Typical mechanical spectra for mushroom‐induced gelation of ewe's milk at different incubation temperature 35°C (a) and 45°C (b). G′, G′′, and complex viscosity are presented with filled, open, and dotted symbols, respectively

The protein gels can be characterized as entangled networks, chemical or physical gels, which mechanical properties of the gels can be determined by frequency sweep experiments (Stading & Hermansson, 1990). Indeed, the mechanical properties of the milk curd are related to network composition, structure, and interactions among molecules within the network (Lucey, Johnson, & Horne, 2003). The mechanical spectra of G′ and G′′ of ficin‐induced gel and mushroom‐induced gel after cooling to 25°C showed a nearly linear relationship over the frequency range 0.01–10 Hz (Figures 3 and 4). In general, the storage modulus was increased as the frequency increases. It can be attributed to the network structure “not relaxing” during the short period as frequency increases. In this case, “relaxing” can be considered a flow of molecules past each other. There is less change seen in G″ with frequency, but note that the scale for G″ is one order of magnitude less than G′. Although, findings revealed that the proteolytic activity of ficin and *P.badius* on ewes’ milk, they showed less G′, G′′, and G* in compared with soft cheese such as UF Feta cheese and Gouda cheese (G*~170 kPa) (Wium & Qvist, 1997). Moreover, the slopes of log (G′/G′′ and G*) were less than that of them. The phase angle (δ) was also reflected the overall viscoelasticity of the gel (Barnes et al., 1989; Steffe, 1996). As frequency increases, the elastic component (G′) increased more than viscous component (G′′) and therefore a reduction in phase angle was happened and exhibited a gel network. As acid milk gels are known to be less viscous in nature than rennet milk gels (van Vliet et al., 1991), the difference in phase angles of two induced gels may to a large extent be, due to special proteolytic activities of ficin and *P. badius*.

The dependency of G′ to frequency can be determined by the constant *n* value as follows:

(1) $$\text{log}\;G^{\prime }=n\text{log}\;\omega \;\text{+K}$$

where G′ is the storage modulus, ω is the oscillation frequency, and k is a constant. The *n* value is considered as an indication of the viscoelastic nature of the gels (Ikeda & Foegeding, 1999). For purely elastic gels, *n* is zero and becomes higher with increasing relative contribution from the viscous component (less elastic). The degree of frequency dependence (*n*) of ficin and mushroom enzyme at different incubation temperatures is presented in Table 2. It can be seen, as the enzyme concentration increased from 1 to 3%, the *n* value was reduced and approached to 0, which indicate the elasticity of gel is governed over the viscous component. Furthermore, by increasing the incubation temperature, the *n* value was reduced and gel strength was improved. In comparison, the more elastic gel was obtained for mushroom enzyme at higher concentrations and temperatures (45°C). Moreover, the *n* value was not significantly different at 1% and 3% ficin enzyme at both incubation periods (*p* < .05). These *n* values, particularly for mushroom enzymes showed a relatively weak physical gel, which have also been observed for β‐lactoglobulin and whey proteins in our previous work (Rafe et al., 2013, 2016). The viscoelastic nature of gels formed by ficin and mushroom enzymes were more close to elastic gel (less *n* value) in comparison with rice bran protein (0.18), which have shown the more contribution of viscous component in gel formation (Rafe et al., 2016).

**Table 2:** The *n* value of log G′‐frequency (0.01–10 Hz) plot for ficin extract and mushroom enzyme‐induced gelation at different incubation temperatures

Incubation Temperature, °C	Ficin enzyme			Mushroom enzyme		
	1%	3%	5%	1%	3%	5%
35	0.166 ± 0.01	0.163 ± 0.01	0.144 ± 0.01	0.171 ± 0.01	0.092 ± 0.01	0.090 ± 0.01
45	0.158 ± 0.02	0.154 ± 0.03	0.132 ± 0.02	0.165 ± 0.01	0.090 ± 0.02	0.083 ± 0.01

### 3.5 Microstructure of ficin and mushroom enzyme‐induced gels

**Figure 5:**
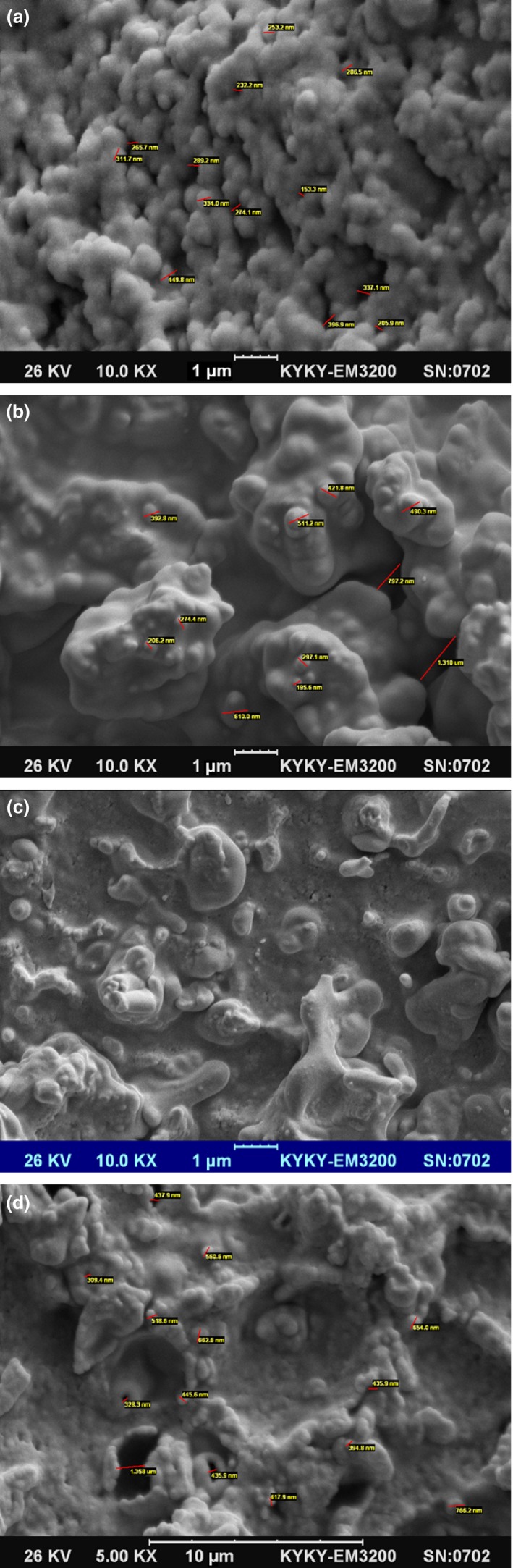
SEM micrographs of induced gelation of ewes’ milk in the presence of ficin extract at 35 (a), and 45°C (b), and *P.badius* extract at 35 (c), and 45°C (d)

Morphological study can give valuable information concerning the size, shape, distribution, and arrangement of protein molecules in the gel structures. Moreover, each gel has a unique microstructure features which reflect the chemical and biological changes occurred during gel formation. Micrograph of gels which were formed in the presence of ficin and *P. badius* enzymes at different incubation temperatures (35 and 45°C) are provided in Figure 5. The SEM results showed that gels induced by ficin have more globular structure than that of mushroom ones. Furthermore, the globular structure was more dominant at lower incubation temperature, which indicates the coagulation temperature has a critical effect on the gel network. On the other hand, by increasing the coagulation temperature, the proteins were denatured faster and had a denser structure. In comparison, it can be found a more uniform and less porous structure in the gels formed by mushroom enzymes. As a result, the protein matrix had more spaces in the ficin‐induced gel and it can be predicted that the mushroom gels would have the high water holding capacity and less synersis. Furthermore, both gels had a continuous structure, which was similarly found in whey proteins (Rafe et al., 2013, 2016; Van den Berg et al., 2007).

The distribution of protein structure was not uniform in the gels formed by *P.badius*, reflecting its less elasticity and softness. Moreover, micrographs of *P.badius* gels had more fusions and folds which indicate greater proteolysis occurred during gelation and there was greater breakdown of protein. Both gels showed a coarser and more compact protein network by increasing the enzyme concentration. Our findings showed that coarsening of the gel network and improving the gel elasticity are parallel events, which are in agreement with previous works (Madadlou, Khosroshahi, & Mousavi, 2005; Wium & Qvist, 1998).

## 4 CONCLUSION

The rheological and structural characteristics of ewe's milk protein in the presence of ficin and *P.badius* were investigated. As ficin extract concentration increases, the more elasticity of gels was obtained. The temperature during incubation and cooling periods has a pronounced effect on the gel elasticity. Similar findings were obtained for mushroom enzyme gels. Therefore, it can be concluded that incubation at 45°C with more ficin/ mushroom enzyme can develop stronger gels. However, *P. badius* gels provide a network with more viscous characteristics and had a softer texture than ficin gels, it may be concluded that induced gels with mushroom had higher moisture and lower protein contents, related to its high proteolytic activity. In contradiction with soft cheeses or rennet milk gels, induced gels showed less proteolytic activity of ficin and *P.badius* on ewes’ milk and therefore a less hard gel was obtained. The mechanical findings confirmed the viscoelasticity of gels, particularly for mushroom enzymes, which showed a relatively weak physical gel. The SEM results showed that gels induced by ficin have more globular structure than that of mushroom ones. Furthermore, the coagulation temperature has a critical effect on the gel network. As a result, the protein matrix had more spaces in the ficin‐induced gel and it can be predicted that the mushroom gels would have the high water holding capacity and less synersis. The distribution of protein structure was not uniform in the gels formed by *P.badius*, reflecting its less elasticity and the hardness. Both gels showed a coarser and more compact protein network by increasing the enzyme concentration. Our findings showed that coarsening of the gel network and improving the gel elasticity are the parallel events.

## CONFLICT OF INTEREST

None declared.
